# Genome Sequencing of *Pleurozium schreberi*: The Assembled and Annotated Draft Genome of a Pleurocarpous Feather Moss

**DOI:** 10.1534/g3.119.400279

**Published:** 2019-07-08

**Authors:** Eric R. A. Pederson, Denis Warshan, Ulla Rasmussen

**Affiliations:** * Department of Ecology, Environment and Plant Sciences, Stockholm University, 106 91 Stockholm, Sweden; † Faculty of Life and Environmental Sciences, University of Iceland, 101 Reykjavík, Iceland

**Keywords:** *Pleurozium schreberi*, Genome sequencing, Comparative genomic, Annotation, Genome assembly

## Abstract

The pleurocarpous feather moss *Pleurozium schreberi* is a ubiquitous moss species which plays a fundamental role in many terrestrial ecosystems, for instance within the boreal forest, the Earth’s largest terrestrial biome, this species plays a significant role in driving ecosystem nitrogen and carbon inputs and fluxes. By hosting dinitrogen (N_2_)-fixing cyanobacteria, the moss-cyanobacteria symbiosis constitutes the main nitrogen input into the ecosystem and by the high productivity and the low decomposability of the moss litter, *P*. *schreberi* contributes significantly to build-up soil organic matter, and therefore long-term C sequestration. Knowledge on *P. schreberi* genome will facilitate the development of ‘omics’ and system’s biology approaches to gain a more complete understanding of the physiology and ecological adaptation of the moss and the mechanisms underpinning the establishment of the symbiosis. Here we present the *de novo* assembly and annotation of *P. schreberi* genome that will help investigating these questions. The sequencing was performed using the HiSeq X platform with Illumina paired-end and mate-pair libraries prepared with CTAB extracted DNA. In total, the assembled genome was approximately 318 Mb, while repetitive elements account for 28.42% of the genome and 15,992 protein-coding genes were predicted from the genome, of which 84.23% have been functionally annotated. We anticipate that the genomic data generated will constitute a significant resource to study ecological and evolutionary genomics of *P. schreberi*, and will be valuable for evo-devo investigations as well as our understanding of the evolution of land plants by providing the genome of a pleurocarpous moss.

*Pleurozium schreberi* (Brid.) Mitt. also known as the red-stemmed feather moss belongs to the order Hypnales, family Hylocomiaceae. *P. schreberi* has a wide global distribution and is one of the most common moss species dominating the ground layer in the boreal forest, sub-alpine and Arctic ecosystems (Nilsson and Wardle 2005; Lindo and Gonzalez 2010). *P. schreberi* serves a variety of key functions in its ecosystem; for instance, *P. schreberi* can host dinitrogen (N_2_)-fixing cyanobacteria, a symbiosis which serve as the major input of nitrogen (N) into boreal forests and thus play a vital role in primary productivity of this N limited ecosystem (Turetsky 2003; Lindo *et al.* 2013). High-resolution secondary ion mass spectrometry verified transfer of fixed N from the cyanobacteria to the moss host, further demonstrating that the symbiosis governs the main N entrance to boreal forests (Bay *et al.* 2013). While, the genetic and genomic of *P. schreberi* symbiotic cyanobacteria have been previously studied (Ininbergs *et al.* 2011; Warshan *et al.* 2016, 2017 and 2018), nothing is known about the moss genomic diversity and gene repertoire needed to form the symbiosis. In particular the genome will be a crucial tool for further studies on the symbiotic interaction with N_2_-fixing cyanobacteria where previous study, have shown the importance of moss secreted signaling molecules to attract the cyanobacteria (Bay *et al.* 2013). Further, the biomass of *P. schreberi* and *Hylocomium splendens* (another ubiquitous feather moss in the boreal forest) can account for up to a third of the total forest productivity (Goodale *et al.* 2002). As boreal forests represent one of the largest terrestrial carbon (C) sinks on Earth, storing approximately 30% of total terrestrial C stocks (Goodale *et al.* 2002; Pan *et al.* 2013) the feather mosses play a significant role in the global C cycles. As such, the response of the feather moss cover to future climate change may influence whether and how the ecosystem will be regulated and the consequences for C sequestration by this biome (Lindo *et al.* 2013). The availability of *P. schreberi* genomic resource will facilitate population genetic study of *P. schreberi* at genomic level using genotyping-by-sequencing approaches. For instance, investigating the link between intraspecific genetic diversity and moss traits variations, such as its physiology, decomposition rate and C accumulation could reveal pattern of local adaptations that might have an impact on N and C cycles. Even though mosses are the second most diverse phylum of land plant with approximately 13,000 species (Goffinet *et al.* 2009), to date only two other moss species had their genome sequenced *i.e.*, *Sphagnum fallax* (DOE-JGI, http://phytozome.jgi.doe.gov/; Shaw *et al.* 2016), and *Physcomitrella patens* (Rensing *et al.* 2008). The latter of which have become a very important model organism (Cove *et al.* 2009).

In this study, we present a draft genome of an axenic pleurocarpous feather moss *P. schreberi*. Indeed, the sequenced draft genome of the moss will be a fundamental resource for future research spanning from evolutionary to ecological aspects.

## Materials and Methods

### Sample collection

**Figure 1:**
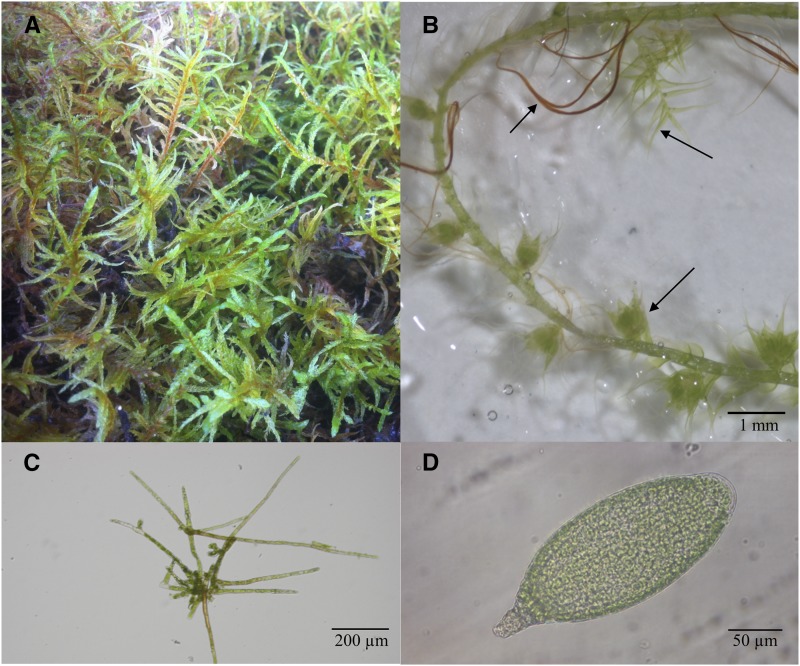
Different growth morphology of *P. schreberi*. (A) Wild type *P. schreberi*. (B) Axenic *P. schreberi* growing on BCD media showing the gametophore, rhizoids, phyllids and reproductive bundles. (C) Protonemal tissue and (D) male gametangia.

*Pleurozium schreberi* is a pleurocarpous moss with typical tissue growth such as leafy gametophores with phyllids, rhizoids and reproductive organs (Figure 1A, B), protonemal tissue (Figure 1C) as well as gametangia (Figure 1D). Intact *P. schreberi* sporophytes were randomly collected June 26, 2016 on Blidö (59°62’20.06”N, 18°90’26.41”E), an island located in the Stockholm archipelago, Sweden. An axenic line was generated by first separating the septa from the sporophyte with forceps and then placing in 99.9% ethanol for 1 min, subsequently moved to 1 min in 5% sodium hypochlorite followed by 4 rounds of rinsing in sterile water. The sterilized spores where placed on BCD media (Cove *et al.* 2009) + 5% sucrose for approximately 2 weeks. Axenic germinated spores were transferred to BCD and placed in an incubator at 24° with constant white light at 30 W/m^2^ to allow the gametophore to growth. Pictures of the *P. schreberi* tissues were taken using the Zeiss Stemi 2000-C Stereo microscope equipped with an AxioCam HR microscope camera (Zeiss, Oberkochen, Germany).

### Genome sequencing and assembly

DNA extraction was performed from gametophores originating from a single spore isolate according to Schlink and Reski (2002) with the following modifications; 10% CTAB was used during the extraction and DNA was re-dissolved in water. After the DNA quality check using the Bioanalyzer 2100 system (Agilent Technologies, Santa Clara, USA), 180bp average fragment size Illumina TruSeq PCR-free library (Illumina, Cambridge, UK), 3kb and 5-8kb insert size Nextera mate-pair libraries (Illumina) were constructed at SciLifeLab (Stockholm, Sweden) following manufacturer’s recommendations. Sequencing proceeded on the Illumina HiSeq X platform, where the Illumina TruSeq PCR-free library and the Nextera mate-pair libraries were sequenced on separate lanes at 2x150bp. Preliminaries *de novo* assemblies were conducted with NouGAT, NGI open universal Genome Assembly Toolbox pipeline (https://github.com/vezzi/NouGAT) using Abyss (Simpson *et al.* 2009), Allpaths (Butler *et al.* 2008), and SOAPdenovo (Luo *et al.* 2012) assemblers with default settings. The quality of the selected assembly was assessed using BUSCO version 3 (Simão *et al.* 2015) with the eukaryota database (release 9) in protein mode. We estimated genome size using k-mer counting of quality and barcode trimmed reads using Jellyfish v2.2.10 (Marçais and Kingsford 2011). K-mer frequency distributions of 21-, 25-, 31-, 41-, 65-mers were generated, then we used findGSE (Sun *et al.* 2017) to estimate genome size and repeat content.

### Repeat content analysis

To identify repeats in the genome assembly, a custom species-specific repeat library was created, using the RepeatModeler package 1.0.8 (Smit 2010) with default parameters. As repeats can be part of actual protein-coding genes, the candidate repeats were vetted against the Uniprot/Swiss-prot proteins set (minus transposons) to exclude any nucleotide motif stemming from low-complexity coding sequences. From the repeat library, identification of repeat sequences present among the genome was performed using RepeatMasker 4.0.3 (Smit *et al*. 2010) and RepeatRunner (Magrane and Consortium 2011).

### Gene prediction

The gene models were carried out using the MAKER pipeline, version 3.01.1 (Cantarel *et al.* 2008; Holt and Yandell 2011). The gene models incorporate *ab initio* gene prediction, homology-based prediction and RNA-seq assisted prediction. Prior to *ab initio* gene prediction, repeat regions of the moss were masked based on repeat annotation results. Homology-based prediction and RNA-seq assisted prediction used proteins sequences collected on the Uniprot database (Magrane and Consortium 2011) selecting those belonging to the Swiss-prot section and nine *P. schreberi* transcriptomes coming from the JGI study ID number Gs0110198. The transcriptome of *P. schreberi* (biosample ID Gb0144502- Gb0144504) had already been assembled by JGI using Rnnotator 3.4.0 (Martin *et al.* 2010) and completed with Velvet 1.2.07 (Zerbino and Birney 2008). The six other transcriptomes (ID Gb0144505- Gb0144510) have been assembled using HISAT2 (version 2.1.0) (Kim *et al.* 2015) and StringTie 1.2.2 (Pertea *et al.* 2015) with default parameters. MAKER *ab initio* training was performed using Augustus (Stanke *et al.* 2008), GeneMark (Besemer and Borodovsky 2005) and SNAP (Bromberg and Rost 2007) with transcripts and high-confidence proteins data. For *ab initio* training purpose, a gene set was created by selecting the best gene models based on i) genes have to be complete (*i.e.*, start/stop codons mandatory); ii) no similarity over 85 is allowed among genes of the set; iii) Annotation Edit Distance (AED) scores have to be inferior to 0.3; iv) genes have to be at a distance of 1,000 bp from each other. In total, 4,264 genes were selected and used for Augustus and SNAP training process. GeneMark has also been trained using splicing sites information of nine transcriptome assemblies. Then, an evidence-guided build was computed by allowing the MAKER software to construct gene models directly from both aligned transcript sequences and reference proteins. Finally, a second round of annotation using MAKER with *ab initio* tools Augustus, SNAP, GeneMark and EvidenceModeler (Haas *et al.* 2008) and evidence build (proteins and transcripts) allowed creating an *ab initio* evidence-driven gene build. After a visual analysis of the *ab initio* evidence-driven gene build, spurious ORFs among the gene structure were discovered in some loci, and fixed using in-house scripts (https://github.com/NBISweden/GAAS). In the case of loci containing proteins and/or transcripts without *ab initio* prediction, the gene models were taken from the evidence build.

### Gene annotation

The functional inference for genes and transcripts were performed using the translated CDS features of each coding transcript. Each predicted protein sequences were blasted against the Uniprot/Swissprot database to retrieve the gene name and the protein function as well as run against InterProscan 5.21-60 (Jones *et al.* 2014) to retrieve functional information from 21 different sources (Table S1).

Gene name inference was performed with the best blast hit approach using the Uniprot reference data set (Kim *et al.* 2015). Only the hits with an e-value inferior to 10E-7 were taken into account. tRNAs have been predicted by using tRNAscan v1.3.1 (Lowe and Eddy 1997) with default settings and other non-coding RNAs by using the RNA family database Rfam (Nawrocki *et al.* 2015) with conserved eukaryotic models only.

### Analyses of potential whole genome duplication in P. schreberi

Whole genome duplication (WGD) events can be detected using synonymous substitution rates (Ks) among pairs of paralogous genes. WGD event(s) within *P. schreberi* were estimated using the FASTKs pipeline (https://github.com/mrmckain/FASTKs). Protein-coding genes were blasted against themselves using an e-value cutoff of 10E-5 and then putative pairs were filtered using FASTKs default parameters. Amino acid sequences for putative paralog pairs were then aligned using MUSCLE v3.8.31 (Edgar 2004), and back translated to CDS using PAL2NAL v14 (Suyama *et al.* 2006). Ks were estimated for the aligned pairs using codeml in PAML v4.8 (Yang 2007) using the same parameters used by McKain *et al.* (2012). All Ks values ≤0.1 were excluded for analysis to avoid the incorporation of allelic variants and to prevent the fitting of a component to infinity, while Ks values >5.0 were removed because of Ks saturation (Vanneste *et al.* 2015). Normal mixture models were estimated for Ks values using the mclust v.5.4.3 (Scrucca *et al.* 2016) in R (v3.5.1). We evaluated mixture models with between one and nine components, and the best fit model was chosen using the Bayesian Information Criterion (BIC).

### Comparative genomic

A comparative genomic analysis was performed on three different moss genomes (*Physcomitrella patens*, *Sphagnum fallax* and *P. schreberi*) using OrthoMCL 2.0.9 (Li *et al.* 2003) with mcl-14-137 (Enright *et al.* 2002) and BLAST+ 2.2.28 (Camacho *et al.* 2009) to infer homologous (both orthologous and paralogous) relationships among a set of protein sequences. The input data used included protein sequences from *P. schreberi*, *P. patens* (v1.39) and *S. fallax* (v0.5). For each moss genome, the CDS were filtered to keep only the longest coding sequence for each gene, and the corresponding sequences were extracted, translated and filtered to retain those larger than 50 amino acids (Table S2). An all-against-all BLASTP analysis of the sequences of these moss species led to the identification of candidate homologs. BLAST hits with an e-value >10E-5 and for which the query and the hit sequence had <50% overlap of their gene length, were excluded. Gene families characterized by the OrthoMCL analysis were processed to infer the presence/absence of the genes along a species tree to provide an evolutionary view of the gene flux in those genomes.

### Data availability

The raw data are deposited in NCBI with SRA accessions numbers; SRR8297981 and SRR8297982 (BioProject accession number PRJNA509035). The BioSample is available with accession number SAMN10578977 at NCBI. The assembled genome is available with the accession number VACF00000000 at NCBI. All of the annotation files are available at github: https://github.com/PycnopodiaD/Pleurozium_schreberi_annotated_genome_files. Supplemental material available at Figshare: https://doi.org/10.6084/m9.figshare.7775951.

## Results and Discussion

### Genome assembly

Concerning the initial genome assemblies of *Pleurozium schreberi*, the Allpaths assembler showed the best assembly statistics (*i.e.*, highest *NG_50_* value and lowest number of scaffolds) compared to assemblies done by Abyss and SOAPdenovo (Table S3). Quality control of the Allpaths assembly including coverage distribution, GC content, contig length and median coverage are shown in Figure S1. Low coverage contigs showed deviation in GC content, which might be a sign of contamination, and therefore they were removed from the assembly (Figure S1C). The total length of the assembly was 318.34 Mb in 2,695 scaffolds with an *NG_50_* score of 204 Kb, and a genomic GC content of 26.4% (Table 1). The repeat regions were estimated to account for 28.42% of the genome (90 Mb; Table 2), and BUSCO analysis captured 90.1% of genes in the eukaryota database with 75.6% complete, 14.5% partial and 9.9% missing genes (Table 3). Even if the genome size and number of repetitive elements in *P. schreberi* appears smaller compared to *P. patens*, they are similar to the estimates of *S. fallax* assembly (genome size of 475.8 and 396 Mb, and percentage of repetitive elements of 57% and 32% for *P. patens* and *S. fallax*, respectively; Shaw *et al.* 2016; Lang *et al.* 2018). When comparing to the k-mer analysis, the predicted genome size and percentage of repetitive elements are reasonably close to our assembly (Table S4). Genome size estimates ranged from 296.9 to 319.4 Mb, and repetitive elements ranged from 12 to 26% of the genome (Table S4). Nevertheless, the length of our assembly is small compared to genome size predicted by flow cytometry that indicated a genome 1.3x – 2.5x larger (ranging from 418 – 809 Mb; Voglmayr 2000; Bainard 2011). A high proportion of repeat sequences in *P. schreberi* genome could result in an underestimation of genome size, but k-mer analysis provides an indication that this was not the case, since the percentage of predicted repeated elements was very similar to the one estimated by our repeat analysis (Table 2 and Table S4). Alternatively, since the BUSCO analysis indicates that our genome assembly was incomplete, the discrepancy observed with the flow cytometry estimates could be partially explained by the fraction of coding sequence missing from our data. Besides, considering that the genome size estimations by flow cytometry originate from gametophyte cells (Voglmayr 2000; Bainard 2011), a most likely explanation to the extreme variation observed in genome sizes seems to be endopolyploidy in gametophyte tissues of *P. schreberi*. In addition, different isolates could have different ploidy levels, particularly in widespread species like *P. schreberi*. This appears to be a common phenomenon in this species and in mosses in general (Bainard and Newmaster 2010). Throughout the assembly of a plant genome many difficulties are incurred (Griesmann *et al.* 2018) and it is therefore not surprising that this assembly is less complete at the first draft stage, though future work should be conducted to improve these assemblies.

**Table 1:** Statistics of *Pleurozium schreberi* genome assembly

Total length (bp)	318,338,550
Number of scaffolds	2,695
Number of uncalled bases (N’s)	98,291,915
Number of N-regions	35,733
NG50 (bp)	204,181
GC-content (%)	26.4

**Table 2:** Repeat statistics by RepeatMasker and RepeatRunner

	RepeatMasker	RepeatRunner
Number of repeats	255,686	484
Total size (kb)	90,223	271.39
Mean size (bp)	352.66	560.73
Percentage of the genome (%)	28.34	0.09

**Table 3:** Statistics of the genome completeness using BUSCO

	Number of BUSCO groups	Percentage
Total BUSCO groups searched	303	100
Complete BUSCOs	229	75.6
Complete Single-Copy BUSCOs	167	55.1
Complete Duplicated BUSCOs	62	20.5
Fragmented BUSCOs	44	14.5
Missing BUSCOs	30	9.9

### Genome annotation

The final annotation of the *P. schreberi* genome from the MAKER annotation pipeline included 15,992 protein-coding genes (Table 4). The functional annotation using InterProscan to detect motifs, domains, signatures, and BLASTP on the Uniprot/Swissprot database resulted in putative function annotation of 13,470 proteins (84.23% of the CDS; Table S5). Further, the best blast hit approach using the Uniprot/Swissprot database assigned a name to 12,295 genes (76.88%; Table S5).

**Table 4:** Coding genes annotation statistics

Description	
Number of protein-coding genes	15,992
Average CDS length (bp)	1,015
Average number of exons per mRNA	5.7
Average exon length (bp)	220
Percentage of the genome covered by:	
Gene	15.90
Exons	6.30
Introns	9.30

### Whole genome duplication in P. schreberi

**Figure 2:**
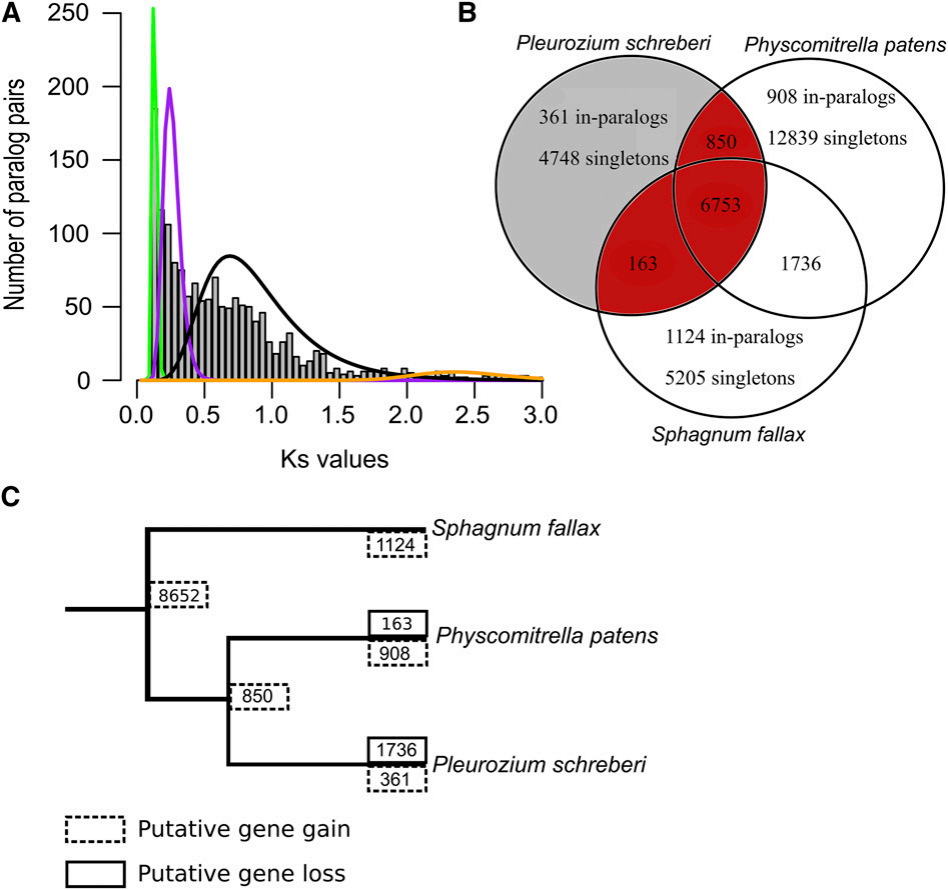
Detection of whole genome duplication in *P. schreberi*, shared and unique orthologs, and gene flux between three moss genomes. (A) Distribution of synonymous substitution rates (Ks) among pairs of paralogous genes within *P. schreberi*. The solid curved lines are the inferred distributions from the mixture model, and colors represent the four component distributions under the model with the highest BIC score. (B) Venn diagram showing shared and unique gene families between moss species. Gene families shared between *P. schreberi*, *P. patens* and/or *S. fallax* are shown in red and the set unique to *P. schreberi* in gray. (C) Phylogenetic relationships between moss species and inferred gene families gain and loss in this lineage. The numbers of gain/loss events are represented by a solid and dotted box, respectively.

Our Ks-based analysis of paralogs was able to detect a peak of Ks values between 0.5 and 2.0, which support one WGD in *P. schreberi* (Figure 2A). Ks analyses conducted on *P. schreberi* and 24 other Hypnales transcriptomes, were unable to detect a clear evidence of WGD event (Johnson *et al.* 2016), but our analysis showed a stronger signal of WGD. When Gaussian distributions were fitted to the Ks values the best fit was of 4 components, but no obvious second large peak of Ks values was observed (Figure 2A). This would have been an indication of a second WGD event in *P. schreberi*, as observed in *P. patens* and *S. fallax* where several rounds of WGD and/or multiple large-scale duplication events have occurred (Devos *et al.* 2016; Lang *et al.* 2018). When considering that the number of protein-coding genes in the genome of *P. schreberi* is 2x smaller than in *P. patens* and 1.7x smaller than in *S. fallax* (32,926 and 26,939 CDS for *P. patens* and *S.fallax*, respectively), the fact that only one WGD event occurred in *P. schreberi* could explain the difference observed.

### Comparative genome analysis

The comparison of gene content of the *P. schreberi* genome with the two other moss genomes available (*P. patens* and *S. fallax*) was conducted to illustrate potential gene flux between the moss species. We found 6,753 gene families shared between the moss species (Figure 2B, Table S2), and 5,109 gene families unique to *P. schreberi* (361 in-paralogs and 4,748 singletons). The comparative analysis suggests a large evolutionary difference between the three species, while Figure 2B shows that *P. schreberi* is sharing nearly 60% of gene families with *P. patens* and/or *S. fallax*, and that the remaining 40% of gene families identified in *P. schreberi* are unique for the species. Besides, it appears that more gene families are shared between *S. fallax* and *P. patens* (1,736 shared gene families) than with *P. schreberi* (Figure 2B). The gene flux analysis over the evolutionary time line for *P. schreberi*, *S. fallax* and *P. patens*, provides some indications to this observation by predicting that most of the gene gain occurred in the most recent common ancestor between the three moss species (8,652 gene families; Figure 2C), and that after the split with *P. patens*, *P. schreberi* gain 361 gene families and loss the 1,736 gene families that are in common between *P. patens* and *S. fallax* (Figure 2C). Nevertheless, this large putative gene loss in the genome might be due to the incompleteness of our draft assembly (Table 3). Moreover, due to the limited number of moss genomes available, this result remains highly speculative especially considering that in the moss phylogeny, *P. schreberi*, *P. patens* and *S. fallax* are evolutionarily quite distant from each other (Liu *et al.* 2019).

### Conclusions

Illumina HiSeq X combining mate-pair and low error rate short-read sequencing enabled *de novo* assembly of the genome of a pleurocarpous moss species, *Pleurozium schreberi*.

Here, we provide a draft assembly that will be useful for long-read sequencing to improve genome scaffolding and completeness of *P. schreberi* genome. Further, a large part of the genes was functionally annotated (84.23%) and preliminaries gene ontology and comparative analyses were undertaken, which will ease future functional genomic studies. Considering that *P. schreberi* occupies a key role in the ecosystem functioning of cold biomes, the data generated provide the genomic resources to deepen our understanding of the physiology of this ecologically important moss species.

## References

[bib1] Bay G., Nahar N., Oubre M., Whitehouse M. J., Wardle D. A., 2013 Boreal feather mosses secrete chemical signals to gain nitrogen. New Phytol. 200: 54–60. 10.1111/nph.12403 23795916

[bib2] Bainard J. D., 2011 Patterns and biological implications of DNA content variation in land plants. Ph.D. Thesis, University of Guelph, Guelph, Ontario, Canada.

[bib3] Bainard J. D., and Newmaster S. G., 2010 Endopolyploidy in Bryophytes: Widespread in mosses and absent in liverworts. J. Bot. 2010: 7.

[bib4] Besemer J., and Borodovsky M., 2005 GeneMark: web software for gene finding in prokaryotes, eukaryotes and viruses. Nucleic Acids Res. 33: W451–W454. 10.1093/nar/gki487 15980510 PMC1160247

[bib5] Bromberg Y., and Rost B., 2007 SNAP: predict effect of non-synonymous polymorphisms on function. Nucleic Acids Res. 35: 3823–3835. 10.1093/nar/gkm238 17526529 PMC1920242

[bib6] Butler J., MacCallum I., Kleber M., Shlyakhter I. A., Belmonte M. K., 2008 ALLPATHS: De novo assembly of whole-genome shotgun microreads. Genome Res. 18: 810–820. 10.1101/gr.7337908 18340039 PMC2336810

[bib7] Camacho C., Coulouris G., Avagyan V., Ma N., Papadopoulos J., 2009 BLAST+: architecture and applications. BMC Bioinformatics 10: 421 10.1186/1471-2105-10-421 20003500 PMC2803857

[bib8] Cantarel B. L., Korf I., Robb S. M., Parra G., Ross E., 2008 MAKER: An easy-to-use annotation pipeline designed for emerging model organism genomes. Genome Res. 18: 188–196. 10.1101/gr.6743907 18025269 PMC2134774

[bib10] Cove D. J., Perroud P.-F., Charron A. J., McDaniel S. F., Khandelwal A., 2009 The moss *Physcomitrella patens*: A novel model system for plant development and genomic studies. Cold Spring Harbor Protoc. 2009: pdb.emo115. 10.1101/pdb.emo115 20147063

[bib54] Devos N., Szövényi P., Weston D. J., Rothfels C. J., Johnson M. G., 2016 Analyses of transcriptome sequences reveal multiple ancient large-scale duplication events in the ancestor of Sphagnopsida (Bryophyta). New Phyt. 211: 300–318. 10.1111/nph.13887 26900928

[bib11] Edgar R. C., 2004 MUSCLE: multiple sequence alignment with high accuracy and high throughput. Nucleic Acids Res. 32: 1792–1797. 10.1093/nar/gkh340 15034147 PMC390337

[bib12] Enright A. J., Van Dongen S., and Ouzounis C. A., 2002 An efficient algorithm for large-scale detection of protein families. Nucleic Acids Res. 30: 1575–1584. 10.1093/nar/30.7.1575 11917018 PMC101833

[bib55] Goffinet B., Buck W. R., and Shaw A. J., 2009 Morphology, anatomy, and classification of the Bryophyta. Bryo. Biol. 2: 55–138.

[bib13] Goodale C. L., Apps M. J., Birdsey R. A., Field C. B., Heath L. S., 2002 Forest carbon sinks in the northern hemisphere. Ecol. Appl. 12: 891–899. 10.1890/1051-0761(2002)012[0891:FCSITN]2.0.CO;2

[bib56] Griesmann M., Chang Y., Liu X., Song Y., Haberer G., 2018 Phylogenomics reveals multiple losses of nitrogen-fixing root nodule symbiosis. Science 361: eaat1743. 10.1126/science.aat1743 29794220

[bib14] Haas B. J., Salzberg S. L., Zhu W., Pertea M., Allen J. E., 2008 Automated eukaryotic gene structure annotation using EVidenceModeler and the Program to Assemble Spliced Alignments. Genome Biol. 9: R7 10.1186/gb-2008-9-1-r7 18190707 PMC2395244

[bib15] Holt C., and Yandell M., 2011 MAKER2: an annotation pipeline and genome-database management tool for second-generation genome projects. BMC Bioinformatics 12: 491 10.1186/1471-2105-12-491 22192575 PMC3280279

[bib16] Ininbergs K., Bay G., Rasmussen U., Wardle D. A., and Nilsson M.-C., 2011 Composition and diversity of *nifH* genes of nitrogen-fixing cyanobacteria associated with boreal forest feather mosses. New Phytol. 192: 507–517. 10.1111/j.1469-8137.2011.03809.x 21714790

[bib17] Johnson M. G., Malley C., Goffinet B., Shaw A. J., and Wickett N. J., 2016 A phylotranscriptomic analysis of gene family expansion and evolution in the largest order of pleurocarpous mosses (Hypnales, Bryophyta). Mol. Phylogenet. Evol. 98: 29–40. 10.1016/j.ympev.2016.01.008 26811877

[bib18] Jones P., Binns D., Chang H.-Y., Fraser M., Li W., 2014 InterProScan 5: genome-scale protein function classification. Bioinformatics 30: 1236–1240. 10.1093/bioinformatics/btu031 24451626 PMC3998142

[bib19] Kim D., Langmead B., and Salzberg S. L., 2015 HISAT: a fast spliced aligner with low memory requirements. Nat. Methods 12: 357–360. 10.1038/nmeth.3317 25751142 PMC4655817

[bib20] Lang D., Ullrich K. K., Murat F., Fuchs J., Jenkins J., 2018 The *Physcomitrella patens* chromosome-scale assembly reveals moss genome structure and evolution. Plant J. 93: 515–533. 10.1111/tpj.13801 29237241

[bib22] Li L., Stoeckert C., and Roos D., 2003 OrthoMCL: identification of ortholog groups for eukaryotic genomes. Genome Res. 13: 2178–2189. 10.1101/gr.1224503 12952885 PMC403725

[bib23] Lindo Z., and Gonzalez A., 2010 The Bryosphere: An integral and influential component of the Earth’s biosphere. Ecosystems (N. Y.) 13: 612–627. 10.1007/s10021-010-9336-3

[bib24] Lindo Z., Nilsson M.-C., and Gundale M. J., 2013 Bryophyte-cyanobacteria associations as regulators of the northern latitude carbon balance in response to global change. Glob. Change Biol. 19: 2022–2035. 10.1111/gcb.12175 23505142

[bib25] Liu Y., Johnson M. G., Cox C. J., Medina R., Devos N., 2019 Resolution of the ordinal phylogeny of mosses using targeted exons from organellar and nuclear genomes. Nat. Commun. 10: 1485 10.1038/s41467-019-09454-w 30940807 PMC6445109

[bib26] Lowe T. M., and Eddy S. R., 1997 tRNAscan-SE: a program for improved detection of transfer RNA genes in genomic sequence. Nucleic Acids Res. 25: 955–964. 10.1093/nar/25.5.0955 9023104 PMC146525

[bib27] Luo R., Liu B., Xie Y., Li Z., Huang W., 2012 SOAPdenovo2: an empirically improved memory-efficient short-read de novo assembler. Gigascience 1: 18 Erratum: 4: 30. 10.1186/2047-217X-1-18 23587118 PMC3626529

[bib28] Magrane M., and Consortium U., 2011 UniProt Knowledgebase: a hub of integrated protein data. Database: The Journal of Biological Databases and Curation 2011: bar009. 10.1093/database/bar009 PMC3070428 21447597

[bib29] Martin J., Bruno V. M., Fang Z., Meng X., Blow M., 2010 Rnnotator: an automated de novo transcriptome assembly pipeline from stranded RNA-Seq reads. BMC Genomics 11: 663 10.1186/1471-2164-11-663 21106091 PMC3152782

[bib30] Marçais G., and Kingsford C., 2011 A fast, lock-free approach for efficient parallel counting of occurrences of k-mers. Bioinformatics 27: 764–770. 10.1093/bioinformatics/btr011 21217122 PMC3051319

[bib31] McKain M. R., Wickett N., Zhang Y., Ayyampalayam S., McCombie W. R., 2012 Phylogenomic analysis of transcriptome data elucidates co-occurrence of a paleopolyploid event and the origin of bimodal karyotypes in Agavoideae (Asparagaceae). Am. J. Bot. 99: 397–406. 10.3732/ajb.1100537 22301890

[bib32] Nawrocki E. P., Burge S. W., Bateman A., Daub J., Eberhardt R. Y., 2015 Rfam 12.0: updates to the RNA families database. Nucleic Acids Res. 43: D130–D137. 10.1093/nar/gku1063 25392425 PMC4383904

[bib33] Nilsson M.-C., and Wardle D. A., 2005 Understory vegetation as a forest ecosystem driver: evidence from the northern Swedish boreal forest. Front. Ecol. Environ. 3: 421–428. 10.1890/1540-9295(2005)003[0421:UVAAFE]2.0.CO;2

[bib34] Pan Y., Birdsey R. A., Phillips O. L., and Jackson R. B., 2013 The structure, distribution, and biomass of the world’s forests. Annu. Rev. Ecol. Evol. Syst. 44: 593–622. 10.1146/annurev-ecolsys-110512-135914

[bib35] Pertea M., Pertea G. M., Antonescu C. M., Chang T.-C., Mendell J. T., 2015 StringTie enables improved reconstruction of a transcriptome from RNA-seq reads. Nat. Biotechnol. 33: 290–295. 10.1038/nbt.3122 25690850 PMC4643835

[bib36] Rensing S. A., Lang D., Zimmer A. D., Terry A., Salamov A., 2008 The *Physcomitrella* genome reveals evolutionary insights into the conquest of land by plants. Science 319: 64–69. 10.1126/science.1150646 18079367

[bib37] Schlink K., and Reski R., 2002 Preparing high-quality DNA from moss (*Physcomitrella patens*). Plant Mol. Biol. Report. 20: 423a–423f. 10.1007/BF02772133

[bib38] Scrucca L., Fop M., Murphy T. B., and Raftery A. E., 2016 mclust 5: Clustering, Classification and density estimation using Gaussian finite mixture models. R J. 8: 289–317. 10.32614/RJ-2016-021 27818791 PMC5096736

[bib39] Shaw A. J., Schmutz J., Devos N., Shu S., Carrell A. A., 2016 Chapter Five - The *Sphagnum* Genome Project: A new model for ecological and evolutionary genomics, pp. 167–187 in Advances in Botanical Research, edited by Rensing S. A. Academic Press Cambridge, Massachusetts.

[bib40] Simão F. A., Waterhouse R. M., Ioannidis P., Kriventseva E. V., and Zdobnov E. M., 2015 BUSCO: assessing genome assembly and annotation completeness with single-copy orthologs. Bioinformatics 31: 3210–3212. 10.1093/bioinformatics/btv351 26059717

[bib41] Simpson J. T., Wong K., Jackman S. D., Schein J. E., Jones S. J., 2009 ABySS: A parallel assembler for short read sequence data. Genome Res. 19: 1117–1123. 10.1101/gr.089532.108 19251739 PMC2694472

[bib42] Smit A. F., Hubley R., and Green P., 1996-2010 Repeat-Masker Open-3.0. http://www. repeatmasker. Org.

[bib43] Stanke M., Diekhans M., Baertsch R., and Haussler D., 2008 Using native and syntenically mapped cDNA alignments to improve de novo gene finding. Bioinformatics 24: 637–644. 10.1093/bioinformatics/btn013 18218656

[bib44] Sun H., Ding J., Piednoël M., and Schneeberger K., 2017 findGSE: estimating genome size variation within human and Arabidopsis using k-mer frequencies. Bioinformatics 34: 550–557. 10.1093/bioinformatics/btx637 29444236

[bib45] Suyama M., Torrents D., and Bork P., 2006 PAL2NAL: robust conversion of protein sequence alignments into the corresponding codon alignments. Nucleic Acids Res. 34: W609–W612. 10.1093/nar/gkl315 16845082 PMC1538804

[bib46] Turetsky M. R., 2003 The Role of bryophytes in carbon and nitrogen Cycling. Bryologist 106: 395–409. 10.1639/05

[bib47] Vanneste K., Sterck L., Myburg A. A., Van de Peer Y., and Mizrachi E., 2015 Horsetails are ancient polyploids: evidence from *Equisetum giganteum*. Plant Cell 27: 1567–1578. 10.1105/tpc.15.00157 26002871 PMC4498207

[bib48] Voglmayr H., 2000 Nuclear DNA amounts in Mosses (Musci). Ann. Bot. (Lond.) 85: 531–546. 10.1006/anbo.1999.1103

[bib49] Warshan D., Bay G., Nahar N., Wardle D. A., Nilsson M.-C., 2016 Seasonal variation in *nifH* abundance and expression of cyanobacterial communities associated with boreal feather mosses. ISME J. 10: 2198–2208. 10.1038/ismej.2016.17 26918665 PMC4989308

[bib50] Warshan D., Espinoza J. L., Stuart R. K., Richter R. A., Kim S.-Y., 2017 Feathermoss and epiphytic *Nostoc* cooperate differently: expanding the spectrum of plant–cyanobacteria symbiosis. ISME J. 11: 2821–2833. 10.1038/ismej.2017.134 28800136 PMC5702739

[bib51] Warshan D., Liaimer A., Pederson E., Kim S.-Y., Shapiro N., 2018 Genomic changes associated with the evolutionary transitions of *Nostoc* to a plant symbiont. Mol. Biol. Evol. 35: 1160–1175. 10.1093/molbev/msy029 29554291 PMC5913679

[bib52] Yang Z., 2007 PAML 4: Phylogenetic analysis by maximum likelihood. Mol. Biol. Evol. 24: 1586–1591. 10.1093/molbev/msm088 17483113

[bib53] Zerbino D. R., and Birney E., 2008 Velvet: algorithms for de novo short read assembly using de Bruijn graphs. Genome Res. 18: 821–829. 10.1101/gr.074492.107 18349386 PMC2336801

